# Ablation of Small Liver Metastases Presenting as Foci of Diffusion Restriction on MRI–Results from the Prospective Minimally Invasive Thermal Ablation (MITA) Study

**DOI:** 10.3390/cancers16132409

**Published:** 2024-06-29

**Authors:** Niek Wijnen, Rutger C. G. Bruijnen, Annelou A. B. Thelissen, Hugo W. A. M. de Jong, Rachel S. van Leeuwaarde, Jeroen Hagendoorn, Guus M. Bol, Maarten L. J. Smits

**Affiliations:** 1 Department of Radiology and Nuclear Medicine, University Medical Center Utrecht, 3584 CX Utrecht, The Netherlands; 2 Department of Endocrine Oncology, University Medical Center Utrecht, 3584 CX Utrecht, The Netherlands; 3 Department of Surgery, University Medical Center Utrecht, 3584 CX Utrecht, The Netherlands; 4 Department of Medical Oncology, University Medical Center Utrecht, 3584 CX Utrecht, The Netherlands; 5 Department of Radiotherapy, University Medical Center Utrecht, 3584 CX Utrecht, The Netherlands

**Keywords:** C-arm CT, colorectal liver metastases, CTHA, DWI, local tumor progression, technical success, small liver metastases

## Abstract

**Simple Summary:**

Liver metastases presenting as small hyperintense lesions on diffusion-weighted imaging pose a therapeutic challenge for conventional thermal ablation, as they are often occult on ultrasound and CT. This study aimed to assess the efficacy of the Hepatic Arteriography and C-Arm CT-Guided Ablation (HepACAGA) technique, a novel approach that integrates C-arm CT hepatic arteriography with C-arm CT-guided navigation, for treating small liver metastases (≤10 mm) detected on MRI. A total of 15 patients with 26 liver metastases were included. The metastases originated predominantly from colorectal cancer (73%), followed by neuro-endocrine tumors (15%), breast cancer (8%) and esophageal cancer (4%). The HepACAGA technique achieved a 100% technical success rate in detecting and ablating the small metastases. After a median follow-up of 9 months, one tumor recurred (4%) which was successfully treated with a subsequent re-ablation. No complications were observed. These findings demonstrate that the HepACAGA technique can effectively ablate subcentimeter liver metastases identified on MRI.

**Abstract:**

Purpose: Liver metastases presenting as small hyperintense foci on diffusion-weighted imaging (DWI) pose a therapeutic challenge. Ablation is generally not possible since these lesions are often occult on ultrasound and CT. The purpose of this prospective study was to assess if small liver metastases (≤10 mm) detected on DWI can be successfully localized and ablated with the Hepatic Arteriography and C-Arm CT-Guided Ablation technique (HepACAGA). Materials and Methods: All consecutive patients with small liver metastases (≤10 mm), as measured on DWI, referred for ablation with HepACAGA between 1 January 2021, and 31 October 2023, were included. Re-ablations and ablations concomitant with another local treatment were excluded. The primary outcome was the technical success rate, defined as the intraprocedural detection and subsequent successful ablation of small liver metastases using HepACAGA. Secondary outcomes included the primary and secondary local tumor progression (LTP) rates and the complication rate. Results: A total of 15 patients (26 tumors) were included, with liver metastases from colorectal cancer (73%), neuro-endocrine tumors (15%), breast cancer (8%) and esophageal cancer (4%). All 26 tumors were successfully identified, punctured and ablated (a technical success rate of 100%). After a median follow-up of 9 months, primary and secondary LTP were 4% and 0%, respectively. No complications occurred. Conclusion: In this proof-of-concept study, the HepACAGA technique was successfully used to detect and ablate 100% of small liver metastases identified on DWI with a low recurrence rate and no complications. This technique enables the ablation of subcentimeter liver metastases detected on MRI.

## 1. Introduction

Image-guided thermal ablation is a well-established local treatment modality for both primary liver tumors and liver metastases. Given its efficacy and minimally invasive nature, thermal ablation is widely implemented for the treatment of unresectable liver metastases [1,2].

The ability to diagnose very small liver metastases has increased over the last few decades due to advances in MRI image quality. MRI is increasingly being added routinely alongside contrast-enhanced CT in the investigation of patients with liver metastases to improve diagnostic accuracy. A recent multicenter, prospective diagnostic accuracy study (CAMINO trial) demonstrated the added value of MRI for patients scheduled for local treatment of colorectal liver metastases (CRLM) [3]. In 92/298 patients (31%), MRI findings (including newly identified small metastases) led to a change in the treatment plan that was initially based solely on contrast-enhanced CT. Additionally, a recent meta-analysis showed that MRI has the highest sensitivity (80%) for detecting subcentimeter liver metastases compared to contrast-enhanced ultrasound (74%), multidetector CT (60%) and PET/CT (16%) [4]. Among the various MRI sequences, diffusion-weighted imaging (DWI) is the most sensitive for detecting liver metastases, with reported sensitivities ranging from 79% to 85% for subcentimeter liver metastases [5,6,7]. 

Unfortunately, advancements in performing image-guided liver ablation have not kept up with improvements in diagnostic sensitivity. Therefore, liver metastases presenting as small hyperintense lesions on DWI can pose a therapeutic challenge for thermal ablation because they are often occult on ultrasound (US) and CT. Most mainstream ablation techniques rely on US or CT for needle placement [8]. When the tumor cannot be visualized, one can rely on anatomical landmarks or the fusion of pre-ablation imaging with real-time imaging. However, these approaches can be inaccurate and/or susceptible to position changes of the tumor or liver between baseline imaging and intraprocedural imaging [9]. Consequently, puncturing small occult liver metastases often results in a ‘semi-blind’ approach, decreasing the chances of successful ablation and increasing the risk of residual or recurrent tumor.

The introduction of CT hepatic arteriography (CTHA) has significantly improved the outcomes of liver ablation [10,11,12,13]. CTHA involves the insertion of a catheter into the hepatic artery and performing CT while intra-arterial contrast is locally administered. Compared to intravenous contrast injection used in conventional CT-guided ablations, intra-arterial contrast in the hepatic artery requires a substantially smaller amount of iodine contrast agent (total amount 10–40 mL vs. 130–180 mL) while providing superior differentiation between the tumor and surrounding tissue. Moreover, CTHA is considered one of the most sensitive imaging methods to detect small liver tumors [14]. It has been demonstrated that lesions as small as 2 millimeters in diameter can be detected with CTHA [15]. Therefore, the integration of hepatic arteriography in thermal ablation shows promise in improving outcomes of ablation of small liver metastases (≤10 mm). 

At our institute, the HepACAGA technique (Hepatic Arteriography and C-Arm CT-Guided Ablation) was developed as a novel technique for liver ablations. It is a variant of CTHA in which C-arm-CTHA and C-arm-guided navigation are combined [16]. The entire procedure takes place in the angiography suite. Similar to CTHA, the outcomes of liver tumor ablation with the HepACAGA technique have shown superior results compared to conventional (US or CT-guided) ablation [17].

The objective of this proof-of-concept study was to evaluate if small liver metastases (≤10 mm) identified on DWI can be successfully localized and ablated using the HepACAGA technique. 

## 2. Methods

### 2.1. Ethical Approval

The data utilized in this study were extracted from the ‘Minimally Invasive Thermal Ablation (MITA)’ study, a prospective registry of patients undergoing thermal ablation of liver tumors at the University Medical Center Utrecht (Utrecht, The Netherlands). Approval for the MITA database was obtained from the local ethical institutional review board (No. 21/709). All included patients provided written informed consent.

### 2.2. Patients

All consecutive patients with liver metastases having a tumor size ≤10 mm in diameter on pre-ablation MRI and referred for treatment with the HepACAGA technique between 1 January 2021, and 31 October 2023, were reviewed for inclusion in this case series. 1 January 2021 marks the moment when the HepACAGA technique became the primary treatment method for liver tumors at our institute. The selection criteria for inclusion were as follows: (1) liver metastases identified as hyperintense foci on diffusion-weighted imaging (DWI); (2) a tumor size ≤10 mm in diameter as measured on DWI; and (3) an MRI scan (including DWI) of the liver within 3 months prior to thermal ablation. Patients were excluded when the patient was referred for re-ablation of a lesion with local recurrence after prior local treatment, or when the ablation was combined with another local treatment. Patients without imaging follow-up were included in the analysis of the technical success of the procedure. When patients presented with both small (≤10 mm) and larger (>10 mm) tumors, only the metastases with a tumor size ≤10 mm were included in the analysis.

### 2.3. Imaging Protocol

MRI scans of the liver comprised T1 (in-phase and out-of-phase), T2, T2 fat-sat, multiphase contrast series and DWI. Lesions were identified and measured on DWI scans with a b-value of 1000 s/mm^2^. Tumor size was calculated for each lesion with a one-dimensional linear measurement of the largest diameter in the axial plane. Imaging follow-up consisted of an initial MRI or CT scan 1 month after the thermal ablation to assess treatment efficacy, complications, and local tumor progression. Thereafter, patients underwent MRI or CT scans every 3–6 months. 

### 2.4. Ablation

**Figure 1:**
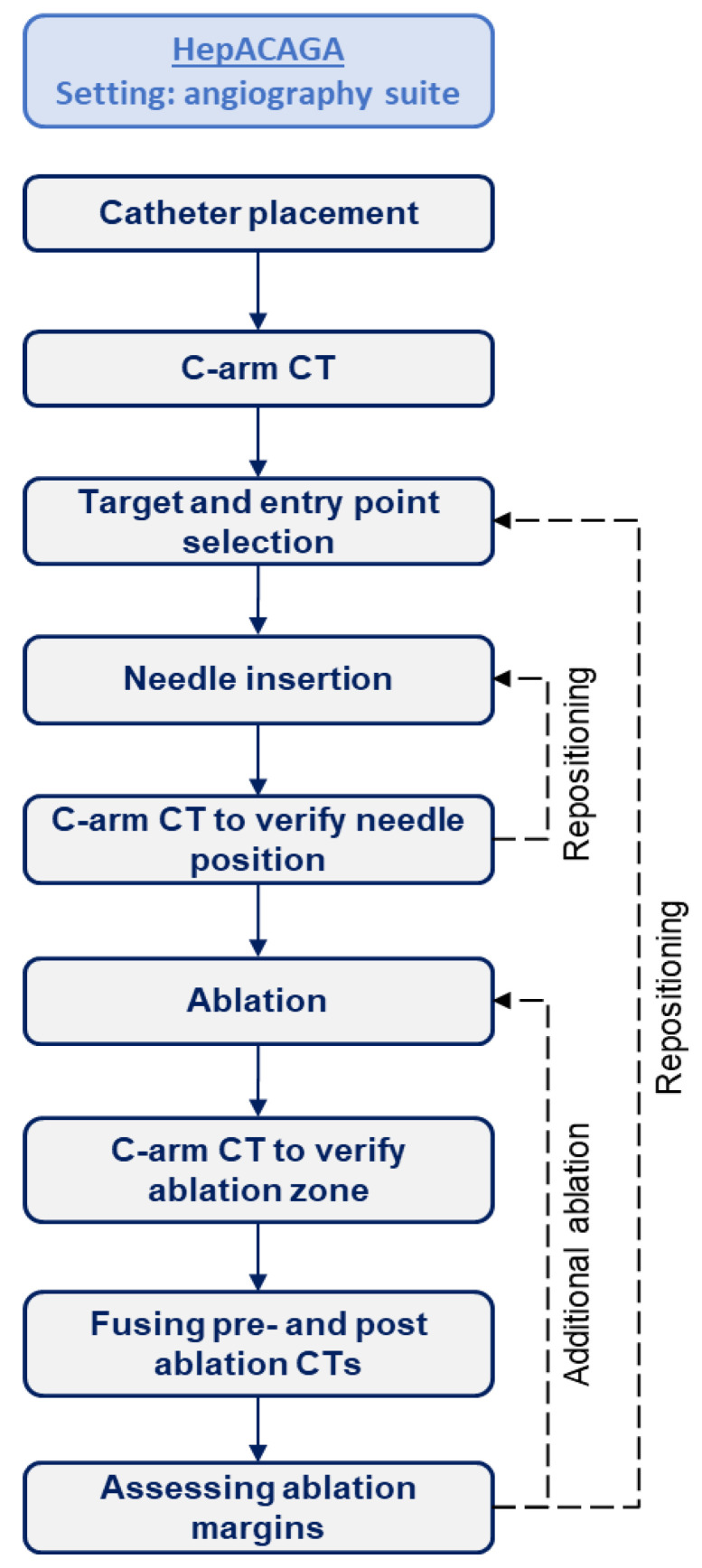
A flowchart of procedural steps involved in the HepACAGA technique.

Tumor ablation was performed according to the HepACAGA technique with the entire procedure performed in the angiography suite [16]. A flowchart outlining the procedural steps is provided in Figure 1. In short, the patient was brought under general anesthesia, the common femoral artery was punctured, and a 5F hydrophilic catheter was placed in the common or proper hepatic artery. In some cases, a 2.7F microcatheter was coaxially advanced more selectively into the right or left hepatic artery. C-arm-CTHA was acquired under apnea with pump injection of small volumes of contrast agent through the catheter into the hepatic artery (Visipaque, GE HealthCare, Chicago, IL, USA) (2:1 dilution with NaCl, flow rate 0.5–2.0 mL/s, total amount 10–40 mL). Apnea was induced by temporarily pausing the ventilator resulting in automatic lung deflation. When the target lesion was identified on C-arm-CTHA, a needle trajectory was planned using the C-arm navigation software (XperGuide, Philips, Best, The Netherlands). The microwave antenna (Emprint^®^ HP, Medtronic, Dublin, Ireland) was then introduced following the planned route under real-time C-arm CT fluoroscopy guidance. Apnea was induced during antenna placement to ensure that the position of the liver and tumor matched the planning C-arm-CTHA. Microwave ablation (MWA) was initiated once the C-arm-CTHA confirmed a correct needle position. A new C-arm-CTHA was performed immediately after thermal ablation was completed. Next, pre- and post-ablation C-arm-CTHAs were semi-automatically fused (using the XperGuide software) to confirm adequate ablation margins. The minimal ablation margin (defined as the shortest distance between the tumor border and the ablation zone boundary in the axial plane) was measured manually, and margins of ≥5 mm were considered adequate. Additional ablation was performed if ablation margins were deemed inadequate. Finally, tract ablation was carried out upon retraction of the antenna.

### 2.5. Study Objectives

The consensus guidelines for the definition of time-to-event endpoints in image-guided tumor ablation were used for the reported outcomes [18].

#### 2.5.1. Technical Success

The primary objective of this study was to determine the technical success rate of ablating small diffusion-restricted liver metastases (≤10 mm) using the HepACAGA technique. Technical success was defined as the intraprocedural detection of the target lesion and its subsequent ablation with adequate margins (minimal margin ≥5 mm). Patient records and radiology reports were examined to assess technical success.

#### 2.5.2. Local Tumor Progression

Primary and secondary local tumor progression (LTP) rates were determined. Primary LTP was defined as the percentage of lesions successfully eradicated after the initial ablation, whereas secondary LTP was defined as the number of tumors that were eventually eradicated after re-ablations. Radiology reports from follow-up imaging were examined to assess LTP following the HepACAGA procedure. Any indication of residual or local recurrence of the tumor at the site of the ablation zone was considered as LTP. 

Local tumor progression-free survival (LTPFS) and 1-year LTPFS were assessed with a per-patient analysis. LTPFS was defined as the duration from the date of ablation until local recurrence of any ablated tumor was observed. Patients were censored at the last follow-up assessment.

#### 2.5.3. Overall Survival

Overall survival (OS) was defined as the duration from the date of ablation until the event of death by any cause. Perioperative mortality, defined as death occurring within 30 days after ablation, was also assessed.

#### 2.5.4. Procedure-Related Characteristics

Patient records were reviewed to identify any intraprocedural or postprocedural complications associated with the HepACAGA procedure in order to assess safety. Complications were evaluated according to the internationally accepted Common Terminology Criteria for Adverse Events (CTCAE), version 5 [19]. Also, the number of needle repositions required, ablation power and ablation duration were examined.

Three time parameters were obtained from anesthesiology reports: (1) the in-room time, defined as the interval between patient arrival and departure; (2) the procedure duration, specified as the interval between the start and end of the procedure; and (3) the per-lesion procedure time, calculated by dividing the total procedure time by the number of treated lesions.

### 2.6. Statistical Analysis

Local tumor progression-free survival and overall survival were assessed using survival analysis. Kaplan–Meier survival curves were generated, with corresponding numbers at risk included. Descriptive statistics were used to summarize the remaining outcomes.

## 3. Results

### 3.1. Patients

Between January 2021 and October 2023, 16 consecutive patients (29 tumors) with small liver metastases (≤10 mm) were treated with the HepACAGA technique. One patient (three tumors) was excluded because these were re-ablations of local recurrences. Consequently, a total of 15 patients with 26 tumors (18 ablation procedures) were included in this case series. Table 1 lists the baseline characteristics of the included patients. The predominant tumor type was colorectal cancer (CRC) (19/26, 73%), followed by neuro-endocrine tumors (4/26, 15%), breast cancer (2/26, 8%) and esophageal cancer (1/26, 4%). The median tumor size, measured as the maximum diameter on DWI, was 7 mm (range 2–10 mm). In 11/15 patients (73%), only small metastases with a tumor size ≤10 mm were ablated, while in 4/15 (27%) patients, both small (≤10 mm) and larger (>10 mm) lesions were ablated (only the subcentimeter lesions were included in this study). 

In 12/15 patients (80%) (22 lesions), US or CT imaging was performed within 6 weeks prior to ablation. The majority of the small metastases (15/22, 68%) were occult on recent US or CT imaging and were only visible as hyperintense foci on DWI. Recent ^18^F-FDG PET/CT imaging was conducted in 2/15 patients (13%) (three lesions) prior to the ablation, with one lesion (33%) being visible on ^18^F-FDG PET/CT.

All included tumors were assigned for ablation using the HepACAGA technique, with none being incidental findings.

### 3.2. Technical Success

**Figure 2:**
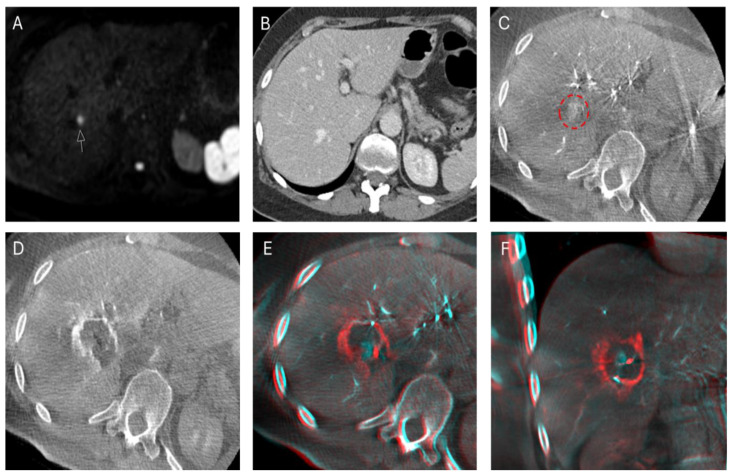
An example of an ablation of a CRLM in segment 8 using the HepACAGA technique (8 min at 120W). (**A**). The CRLM is visible on DWI (white arrow) (size: 9 mm); (**B**). The same lesion is not visible on a CT scan 28 days prior to DWI; (**C**). Intraprocedural detection of the lesion with C-arm-CTHA (red circle); (**D**). The ablation zone depicted with C-arm-CTHA immediately after ablation; (**E**,**F**). Pre- and post-ablation C-arm-CTHAs are fused using the XperGuide software in the axial and coronal planes, showing adequate ablation margins.

**Figure 3:**
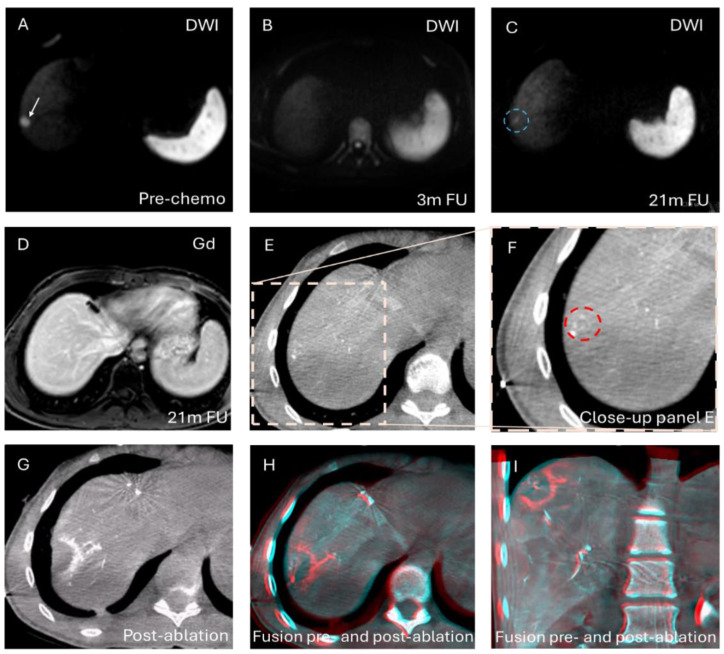
An example of an ablation of a reappearing CRLM that initially disappeared after systemic therapy induction (3 min at 75W). (**A**). The lesion is visible on DWI (white arrow) prior to chemotherapy (FOLFOXIRI-B) induction; (**B**). The lesion disappeared at 3 months follow-up (FU); (**C**). The lesion reappeared as a small diffusion-restricted focus (blue circle) at 21 months follow-up (size: 4 mm); (**D**). The same lesion is barely visible on gadolinium-enhanced MRI (Gd); (**E**,**F**). The lesion is identified on intraprocedural C-arm-CTHA (red circle); (**G**). The ablation zone depicted with C-arm-CTHA immediately after ablation; (**H**,**I**). Pre- and post-ablation C-arm-CTHAs are fused using the XperGuide software in the axial and coronal planes, showing adequate ablation margins.

The HepACAGA procedure was successfully performed in all 26 tumors, resulting in a technical success rate of 100%. This indicates that all 26 tumors were successfully identified using intraprocedural C-arm-CTHA, and subsequent ablation was performed with adequate ablation margins. An example of a HepACAGA procedure of a small colorectal liver metastasis (CRLM) is provided in Figure 2. Another example, showing the ablation of a reappearing CRLM that initially disappeared after systemic therapy induction, is shown in Figure 3.

### 3.3. Local Tumor Progression

One patient (two tumors) had no imaging follow-up data and therefore could not be included in the analysis of local tumor progression. The median imaging follow-up period of the remaining 14 patients was 9 months (range 2–34). MRI alone was used for follow-up in 1/14 patients (7%), while a combination of MRI with CT or ^18^F-FDG PET/CT was conducted in 13/14 patients (93%). In 5/14 patients (36%), new liver tumors were identified outside the ablated liver region during follow-up imaging. These new lesions were treated with local therapy (either thermal ablation or partial hepatectomy) in four patients, while one patient received systemic therapy (chemotherapy).There was local tumor progression in 1/14 patients (7%) and 1/24 ablated tumors (4%). The recurrent tumor was successfully re-ablated with HepACAGA and did not recur again. Therefore, the primary (per-tumor) LTP was 4% and the secondary LTP was 0%.

**Figure 4:**
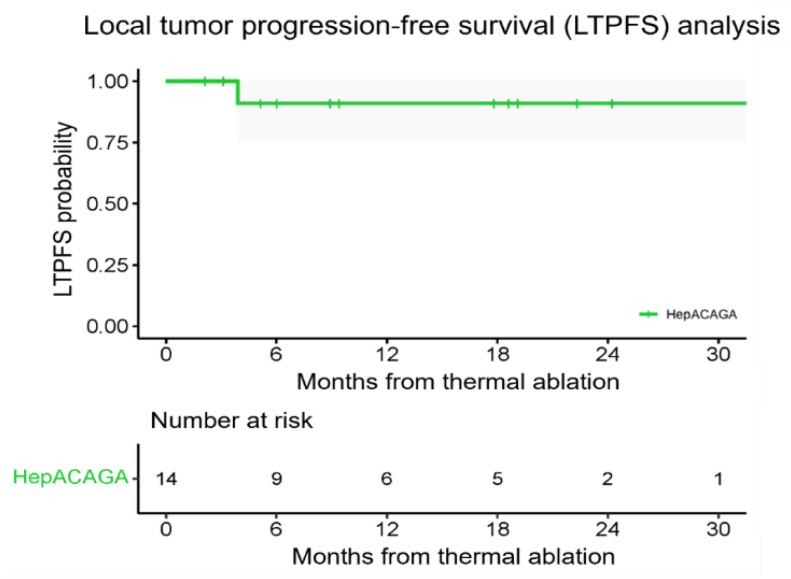
A Kaplan–Meier survival curve illustrating the local tumor progression-free survival (LTPFS) with 95% CI. The number at risk corresponds to the number of patients present at each time point.

The median LTPFS was not reached (Figure 4). The 1-year LTPFS was 91% (95% CI: 0.754–1).

There were no cases of perioperative mortality. A total of 2/14 patients (14%) died during follow-up. The 1-year OS was 93% (95% CI: 0.803–1). The OS Kaplan–Meier survival curve is provided in the supplementary materials (Figure S1).

### 3.4. Procedure-Related Outcomes

During follow-up, no procedure-related mortality or complications were observed, resulting in a complication rate of 0% (Table 2). MWA antenna placement was successful in the first attempt in 15/18 procedures (83%), a single reposition was necessary in 2/18 procedures (11%) and two repositions were necessary in 1/18 procedures (6%). The median ablation power was 100W (range 75–150W), with a median ablation duration of 4.5 min (range 2.5–12.5 min).

The median in-room time was 137 min (range 98–178), the median procedure time was 91 min (range 62–126) and the median per-lesion procedure time was 62 min (range 30–104).

## 4. Discussion

This study showed that small liver metastases (≤10 mm) detected on DWI could be successfully localized and ablated using the HepACAGA technique. Technical success was achieved in all 26 lesions (100%) without any procedure-related complications. One lesion recurred after initial ablation (the primary LTP rate was 4%), which was successfully eradicated with a subsequent re-ablation (the secondary LTP rate was 0%).

Newly detected small liver metastases that are visible as hyperintense foci on DWI (often occult on US or CT) can pose a clinical challenge at multidisciplinary tumor boards. Until recently, the approach in our institute was, in many cases, to wait and scan until the tumor was large enough to be visible on US or CT because there were no imaging techniques available to localize these lesions during local treatment. However, delaying treatment poses a risk of disease progression [20]. With the implementation of CTHA in ablation procedures, intraprocedural visualization and ablation of subcentimeter liver metastases became feasible. Therefore, the introduction of the HepACAGA technique at our institute enabled the ablation of small liver lesions without the need to await tumor growth.

In addition to the implementation of CTHA for facilitating the ablation of occult liver metastases, other studies have investigated the efficacy of intraprocedural real-time ^18^F-FDG PET/CT as image guidance for the ablation of FDG-avid lesions that are occult on CT [21,22]. PET/CT combines anatomical details (CT) with metabolic information (^18^F-FDG PET), allowing for precise localization and targeted ablation of hypermetabolic tumors without the need for repeated contrast injections [23]. PET/CT-guided ablation may be less effective for small occult liver metastases (≤10 mm) due to its limited resolution.

Although technically feasible, this study has not investigated whether early treatment of small metastases is actually beneficial. Small metastases can be broadly categorized into three groups: (1) de-novo oligometastatic disease (OMD), indicating a first-time diagnosis of OMD; (2) repeat OMD, signifying a previous history of OMD; and (3) induced OMD, denoting a history of polymetastatic disease [24]. For the first two groups, there is little controversy about performing local treatment if metastases are confined to the liver and the number of liver metastases is low. There is less damage to non-tumorous tissue if a lesion is ablated while still small (reducing the risk of complications) [25]. It also has been demonstrated that the risk of recurrence after ablation rises as the tumor size increases [1]. However, for induced OMD, one may question the effectiveness of local treatment for metastases that recur after systemic treatment cessation, depending on the treatment interval. The aim of local therapy for induced OMD may be to restore responsiveness to current systematic therapy by ablating insensitive metastases [24]. However, in cases where previous metastases reappear one by one, there is no clear evidence on whether it is best to treat these metastases individually with local therapy, revert to systemic treatment options or await tumor growth before initiating treatment [26]. Further investigation is required to clarify this issue.

In this study, all included lesions were successfully detected and ablated using the HepACAGA technique (the technical success rate was 100%), and no procedure-related complications were observed. All lesions, including the tumor with a maximum diameter of 2 mm (as measured on DWI), were identified using C-arm-CTHA. Lesion sizes were measured on baseline MRI because the decision of whether a patient could undergo ablation or not was based on this MRI. Using intraprocedural C-arm-CTHA, some lesions appeared larger than when measured on baseline MRI. It remains uncertain whether this discrepancy represents actual interval tumor growth, is due to differences in imaging techniques, or both.

Local tumor progression was observed in one lesion after initial ablation (the primary LTP rate was 4%). This recurrence can be explained by a relatively short ablation duration and power (3 min at 75W) resulting in small ablation margins. After re-ablation in a second session, this lesion did not recur again (the secondary LTP rate was 0%). At our institute, we now apply longer ablation durations and a higher power setting to prevent these recurrences. Complete coverage of the tumor by the ablation zone with adequate ablation margins (a minimal margin of at least 5 mm) is presumably the most critical factor for achieving local tumor progression-free survival [27]. A recent meta-analysis of 21 studies emphasized this, showing that ablations with margins <5 mm had a 3.6 times higher risk of local tumor progression compared to ablations with margins ≥5 mm [28].

Besides the increased freedom in angulation, using the C-arm CT for image guidance offers additional benefits for institutions without a hybrid angiography-CT room. The entire procedure can be performed in the angiography suite, eliminating the need to transfer patients between the angiography suite (for catheterization) and the CT room (for ablation). This reduces the risk of catheter dislodgement and sterile field contamination. Additionally, this approach addresses the logistical challenges of reserving both rooms and potentially two teams of technicians or nurses. Performing the ablation in the angiography suite instead of on CT can also be advantageous in case of post-ablation hemorrhage, since embolization can be performed without any delay [16]. Also, one can choose to combine ablation and (chemo-)embolization when working in the angiography suite.

However, the HepACAGA technique also has several limitations. A fundamental drawback of CTHA is the small risk of puncture-site bleeding or infection associated with the catheterization. However, none of the included patients experienced such complications. Another limitation is that general anesthesia was used for all procedures. General anesthesia was used to induce apneas during C-arm CTs and antenna placement. Furthermore, placement of the antenna under real-time fluoroscopy results in additional radiation exposure to the operator.

A limitation of this study is the fact that the treated lesions were very small and there was no histopathological proof that these were indeed metastases. However, diffusion restriction is an essential imaging characteristic for differentiating between benign and malignant focal liver lesions. The sensitivity of DWI (79–85%) for subcentimeter liver metastases is significantly higher than that of T2 weighted imaging (27–44%) or contrast-enhanced CT (43–57%) [6,7]. Also, the detection of new liver lesions exhibiting diffusion restriction in patients with a history of liver metastases strongly indicates their malignancy. 

Another drawback is that not all patients underwent recent CT and US imaging prior to treatment, making it unclear how many lesions in the entire study population were occult on these modalities. This reflects clinical practice, where tumor boards often have to assess whether local treatment is feasible based on the MRI findings. 

Other limitations of this study are the limited number of patients, heterogeneity in primary tumor types, and lack of a control group, which may have influenced the reliability and generalizability of this study. However, this study served as a proof-of-concept to assess the feasibility and efficacy of ablating small liver metastases (≤10 mm) with the HepACAGA technique. 

Another drawback is the limited median follow-up time (9 months, range 2–34 months). Consequently, some patients are still at risk of developing LTP. However, the majority of LTPs are detected within the first three to nine months after ablation [29]. Further studies, preferably disease-specific, will be needed to validate this study’s findings and to investigate if early local treatment of these liver metastases leads to meaningful benefits for these patients given the high rate of out-of-field recurrences during follow-up.

## 5. Conclusions

In conclusion, HepACAGA was demonstrated to be an effective and safe technique enabling the thermal ablation of small occult liver metastases (≤10 mm). Further research with larger cohorts and longer follow-up periods is necessary to validate these initial findings and to determine the broader clinical benefits of early local treatment for such lesions.

## Figures and Tables

**Table 1:** Baseline characteristics.

**Patient-Related Characteristics**		*n* = 15
Sex, *n* (%)	Male	9 (60)
	Female	6 (40)
Age (years), median (range)		56 (16–84)
BMI (kg/m^2^), median (range)		25 (16–32)
ASA, *n* (%)	1	3 (20)
	2	6 (40)
	3	6 (40)
**Tumor-Related Characteristics**		*n* = 26
Primary tumor type, *n* (%)	CRC	19 (73)
	NET	4 (15)
	Breast cancer	2 (8)
	Esophageal cancer	1 (4)
Diameter on DWI (mm), median (range)		7 (2–10)
		*n* = 15
Patients with only small (≤10 mm) metastases, *n* (%)		11 (73)
Patients with both small (≤10 mm) and larger (>10 mm) metastases, *n* (%)		4 (27)
**Pre-Treatment Imaging**		*n* = 15
Patients with recent US or CT imaging prior to ablation, *n* (%)		12 (80)
		*n* = 22
Lesions only visible on DWI (occult on recent US or CT imaging prior to ablation), *n* (%)		15 (68)

ASA = American Society of Anesthesiologists; BMI = Body Mass Index; CRC = Colorectal Cancer; DWI = Diffusion Weighted Imaging; NET = Neuro-endocrine Tumors; US = Ultrasound.

**Table 2:** Procedure-related outcomes.

**Technical Success**		*n* = 26
Per-lesion technical succes, *n* (%)		26 (100)
**Complications**		*n* = 18
Complicated procedures, *n* (%)		0 (0)
**Ablation Parameteres**		
Antenna reposition, *n* (%)	0	15 (83)
	1	2 (11)
	2	1 (6)
Power (Watt), median (range)		100 (75–150)
Ablation duration (min), median (range)		4.5 (2.5–12.5)
**Time Parameters**		
In-room time (min), median (range)		137 (98–178)
Procedure duration (min), median (range)		91 (62–126)
Per-lesion procedure time (min), median (range)		62 (30–104)

## Data Availability

The data presented in this study are available upon request from the corresponding author.

## Supplementary Materials

The following supporting information can be downloaded at https://www.mdpi.com/article/10.3390/cancers16132409/s1, Figure S1: Overall survival Kaplan–Meier curve.
