# Supplementary material for: Ablation of Small Liver Metastases Presenting as Foci of Diffusion Restriction on MRI–Results from the Prospective Minimally Invasive Thermal Ablation (MITA) Study
Source: Cancers (Basel). 2024 Jun 29;16(13):2409. doi: 10.3390/cancers16132409 (PMC11240348; doi:10.3390/cancers16132409)
Supplement: Supplementary file 1 [file cancers-16-02409-s001.zip › cancers-3064664-supplementary.pdf]

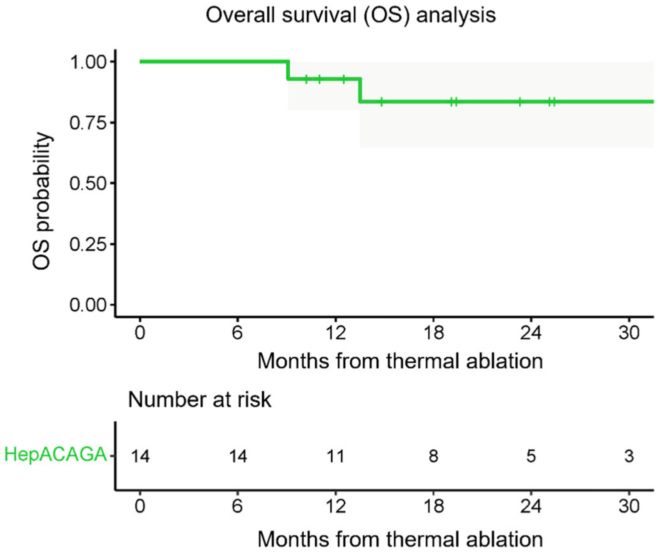

Figure S1. Kaplan-Meier curve illustrating the overall survival (OS) with 95% CI. The number at risk corresponds to number of patients present at each time point.
